# Large variations of oxygen delivery in self-inflating resuscitation bags used for preoxygenation - a mechanical simulation

**DOI:** 10.1186/s13049-021-00885-3

**Published:** 2021-07-19

**Authors:** Sven Grauman, Joakim Johansson, Thomas Drevhammar

**Affiliations:** 1 Department of Anaesthesia and Intensive Care, Östersund Hospital, Region of Jämtland Härjedalen, 83183 Östersund, Sweden; 2 Department of Surgical and Perioperative Sciences, Umeå University, 90187 Umeå, Sweden; 3 Department of Women’s and Children’s Health, Karolinska Institutet. Tomtebodavägen 18A, 17177 Stockholm, Sweden

**Keywords:** Self-inflating resuscitation bags, BVM, Oxygen delivery, Preoxygenation, Emergency anaesthesia

## Abstract

**Background:**

Self-Inflating Resuscitation Bags (SIRB) are common and essential tools in airway management and ventilation. They are often used in resuscitation and emergency anaesthesia outside the operating theatre. There is a common notion that all SIRBs applied with a tight sealed mask will deliver close to 100 % oxygen during spontaneous breathing. The aim of the study was to measure the oxygen delivery of six commonly used SIRBs in a mechanical spontaneous breathing adult *in vitro* model.

**Methods:**

Three SIRBs of each of the six models were evaluated for oxygen delivery during simulated breathing with an adult mechanical lung. The test was repeated three times per device (54 tests in total). The breathing profile was fixed to a minute volume of 10 L/min, a tidal volume of 500 mL and the SIRBs supplied with an oxygen fresh gas flow of 15 L/min. The fraction of delivered oxygen (FDO_2_) was measured over a three-minute period. Average FDO_2_ was calculated and compared at 30, 60 and 90 s.

**Results:**

At 90 s all models had reached a stable FDO_2_. Average FDO_2_ at 90 s; Ambu Oval Plus 99,5 %; Ambu Spur II 99,8 %; Intersurgical BVM Resuscitator 76,7 %; Laerdal Silicone 97,3 %; Laerdal The Bag II 94,5 % and the O-Two Smart Bag 39,0 %. All differences in FDO_2_ were significant apart from the two Ambu models.

**Conclusions:**

In simulated spontaneous breathing, four out of six (by Ambu and Laerdal) Self-Inflating Resuscitation Bags delivered a high fraction of oxygen while two (Intersurgical and O-two) underperformed in oxygen delivery. These large variations confirm results reported in other studies. It is our opinion that underperforming Self-Inflating Resuscitation Bags might pose a serious threat to patients’ health if used in resuscitation and anaesthesia. Manufacturers of Self-Inflating Resuscitation Bags rarely provide information on performance for spontaneous breathing. This poses a challenge to all organizations that need their devices to deliver adequate oxygen during spontaneous breathing.

## Background

Self-Inflating Resuscitation Bags (SIRBs), also known as Bag-Valve-Mask devices (BVMs) or Manual Resuscitators, are common and essential tools in airway management and ventilation. They are mainly used in resuscitation and emergency anaesthesia outside the Operating Theatre (OT) and Intensive Care Unit (ICU), including the prehospital arena. SIRBs are used for positive pressure ventilation in patients with insufficient breathing but also for preoxygenation of the spontaneously breathing. For patients undergoing anaesthesia with no access to ventilators or anaesthetic machines there are few practical alternatives to a SIRB [1–3].

Preoxygenation effectiveness is affected by a compound of several factors including, but not limited to: patient factors (age, anatomy, habitus, pathology, breathing pattern), situational factors (patient position, timing, altitude), operator factors (skills and knowledge) and technical factors (oxygen delivery device characteristics, positive end-expiratory pressure (PEEP), oxygen fresh gas flow rate) [1, 4, 5]. Some of these factors can be optimized by skill, conscious handling and timing, whereas others cannot be changed. The characteristics of the oxygen delivery device are generally fixed to the construction of the specific device.

The fraction of delivered oxygen (FDO_2_) from a device corresponds to the maximum inhaled fraction of oxygen (FiO_2_) and subsequently the fraction of alveolar oxygen achieved in a subject [4]. Thus, a low FDO_2_ directly translates to a suboptimal preoxygenation effect. Clinically, end-tidal oxygen concentration (EtO_2_) can be used as a measurement that reflects preoxygenation efficacy. Clinical recommendations for preoxygenation in anaesthesia is to target an EtO_2_ of at least 0.85–0.9 [4–6]. EtO_2_ can never be higher than FDO_2_ or FiO_2_; making an FDO_2_-capability close to 1.0 paramount for achieving a successful preoxygenation.

The gold standard for preoxygenation is a tight sealed mask connected to an anaesthetic machine, with close to an FiO_2_ of 1.0 [1, 2]. Outside of the OT and ICU the most common devices used for preoxygenation are SIRBs, free-flow oxygen masks, flow-dependent Mapelson systems or high-flow nasal cannulas [1–3, 7]. The ability to manually ventilate apnoeic patients and limited oxygen supply typically makes a SIRB the preferred choice [3].

Previous studies comparing different types of devices for preoxygenation in spontaneously breathing adults have come to conflicting results and different conclusions [2, 7–9]. There is limited information from manufacturers about their device’s performance for this usage. Despite this, there is a common misconception that all SIRBs applied with a tight sealed mask will deliver close to an FiO_2_ of 1.0 during spontaneous breathing [10]. Several studies of varying designs in the last 30 years have highlighted that SIRBs differ markedly in their ability to deliver oxygen to the spontaneously breathing adult [10–14].

Our study revisited the research question of FDO_2_ performance of SIRBs and evaluated six common models on the Scandinavian market. A mechanical lung simulation was used to standardize testing, allowing tests of multiple devices of each model.

The aim of the study was to measure the oxygen delivery of six SIRB models in an adult mechanical spontaneous breathing *in vitro* model.

## Methods

Six models of SIRBs, two reusable and four disposable, were evaluated in a laboratory setting. The characteristics of the different tested SIRBs are presented in Table 1.

**Table 1:** Device presentation and characteristics

Model (model number)	Manufacturer	Bag volume (mL)	Reservoir volume (mL)	Valve type	Expiratory valve	Reusable	Other
Silicone Resuscitator (87005033)	Laerdal	1600	2600	Duck	Disc	Yes	
The Bag II (845141)	Laerdal	1650	2900	Duck	Disc	No	
Oval Plus Silicone Resuscitator (470016000)	Ambu	1546	1500	Disc	NA	Yes	
Spur II (325001000)	Ambu	1547	2600	Disc	NA	No	
BVM Resuscitator (7152000)	Intersurgical	1500	Not stated	Duck	No	No	
Smart Bag MO (01BM3201-MO-Cs)	o_two	1700	1700	Duck	No	No	Tested with SMART-valve disabled^a^

*NA *Not applicable

^a^The SMART-valve limits the pressure/flow to the patient when providing positive pressure ventilation

Three devices of every model were tested for FDO_2_ performance. Each device was tested three times, amounting to nine test sequences per model and 54 sequences in total. The test sequence was conducted with a fixed breathing pattern consisting of a sine waveform with a respiratory rate (RR) of 20 per minute, a tidal volume (TV) of 500 mL and an inspiratory/expiratory ratio of 1:2.

An ASL 5000 mechanical lung simulator (IngMar Medical, Pennsylvania, USA) was used to simulate the spontaneous breathing of an adult. A VT 650 Gas Flow Analyser (Fluke, Washington, USA), used for continuous oxygen sampling, was connected in line between the flow port of the lung machine and the SIRB being tested. A Clear-Guard 3 breathing filter (Intersurgical, Berkshire, UK) was connected between the gas analyser and the SIRB to protect the machines from particle contamination. The oxygen inlet of the SIRB to be tested was connected to 15 L/min of oxygen from a wall outlet and the reservoir and bag filled with oxygen before connection to the breathing circuit. The lung machine was breathing room air through the gas flow analyser before each test until a steady state of FiO_2_ of 0.21 (+/- 0.001) was achieved. Oxygen sampling was recorded 15 s before the SIRB was connected and went on for 180 s in total with a sampling interval of one second. Before each new model was tested the gas flow analyser was calibrated according to the manufacturer’s instructions and the oxygen wall output flow was calibrated to 15 L/min (+/- 0.01 L/min) with a Defender 510 (MesaLabs, California, USA) dry gas meter.

The start of each breath was manually aligned, and average delivered oxygen concentration was calculated for the nine datasets of each tested model. The means with confidence intervals are presented graphically. ANOVA were used to compare means at 30, 60 and 90 s, including post hoc testing with Bonferroni correction for multiple comparisons. These means were calculated for five seconds (-2 to + 2 s) for each model (total *n* = 54). Statistical significance was set to *p* < 0.05.

## Results

**Fig. 1:**
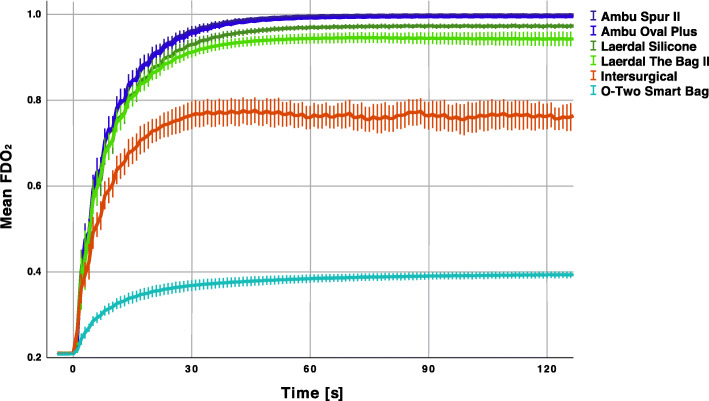
Delivered oxygen during simulated spontaneous breathing. Mean delivered oxygen for the nine recordings of each model. Error bars represent 95 % CI

The FDO_2_ performance of the different SIRBs are shown in Fig. 1. The differences between models were significant at the predefined times 30, 60 and 90 s and is presented in Table 2.

**Table 2:** Average delivered oxygen per model at 30, 60 and 90 s during simulated spontaneous breathing

	Time 30 s O_2_ [%]		Time 60 s O_2_ [%]		Time 90 s O_2_ [%]	
**Ambu Oval Plus**	95.4 (95.1–95.8)^a^		99.2 (99.1–99.2)^a^		99.5 (99.4–99.5)^a^	
**Ambu Spur II**	96.1 (95.7–96.5)^a^		99.6 (99.5–99.7)^a^		99.8 (99.8–99.9)^a^	
**Intersurgical**	76.5 (75.3–77.6)		76.3 (75.3–77.4)		76.7 (75.5–77.9)	
**Laerdal Silicone**	92.9 (92.4–93.4)		96.9 (96.7–97.1)		97.3 (97.1–97.5)	
**Laerdal The Bag II**	91.2 (90.7–91.7)		94.4 (94.0-94.9)		94.5 (94.0–95.0)	
**O-Two Smart Bag**	36.8 (36.4–37.3)		38.4 (38.1–38.8)		39.0 (38.7–39.3)	

System averages were calculated for 5 s (-2 to + 2 s) for the nine recordings of each model (total *n* = 54). All differences, for each time set, were statistically significant apart from the two Ambu systems (^a^). Means (95 % CI)

## Discussion

Four out of six (by Ambu and Laerdal) SIRBs performed well in delivering high and sufficient FDO_2_. One (Intersurgical) of the disposable devices performed markedly worse than the first four, achieving an FDO_2max_ that is below what is needed to achieve an EtO_2_ > 0.85 in preoxygenation. The last disposable model (O-Two) performed so poorly that it presents an even greater risk when used for delivering oxygen to a spontaneously breathing patient.

### Technical considerations

**Fig. 2:**
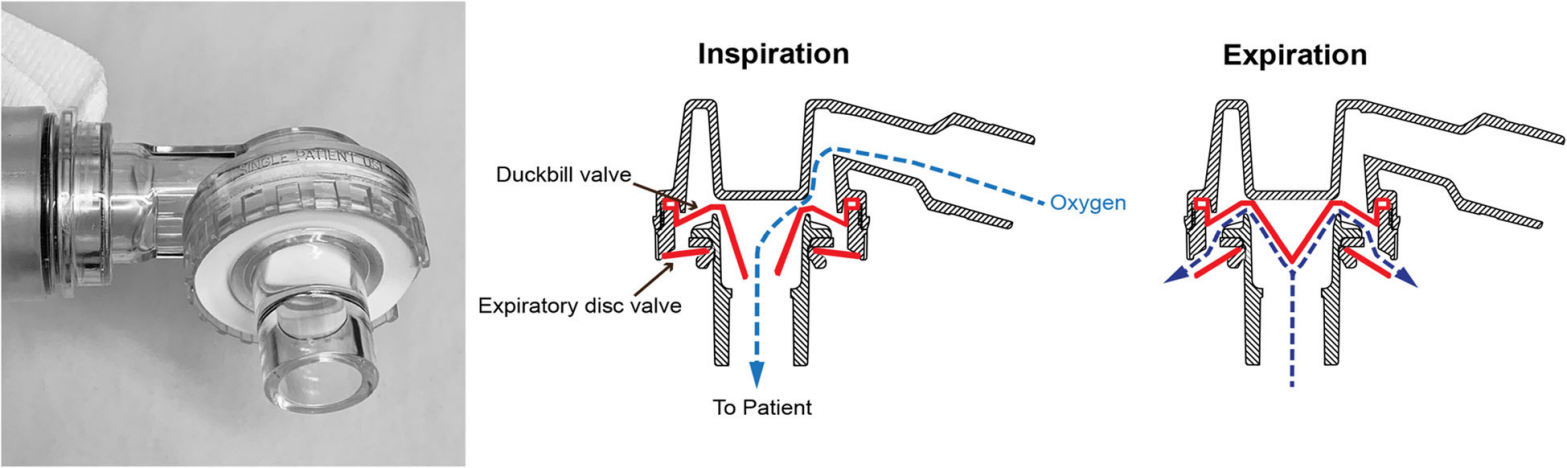
The duckbill valve (Laerdal). Drawing is used and modified with permission from www.laerdal.com

**Fig. 3:**
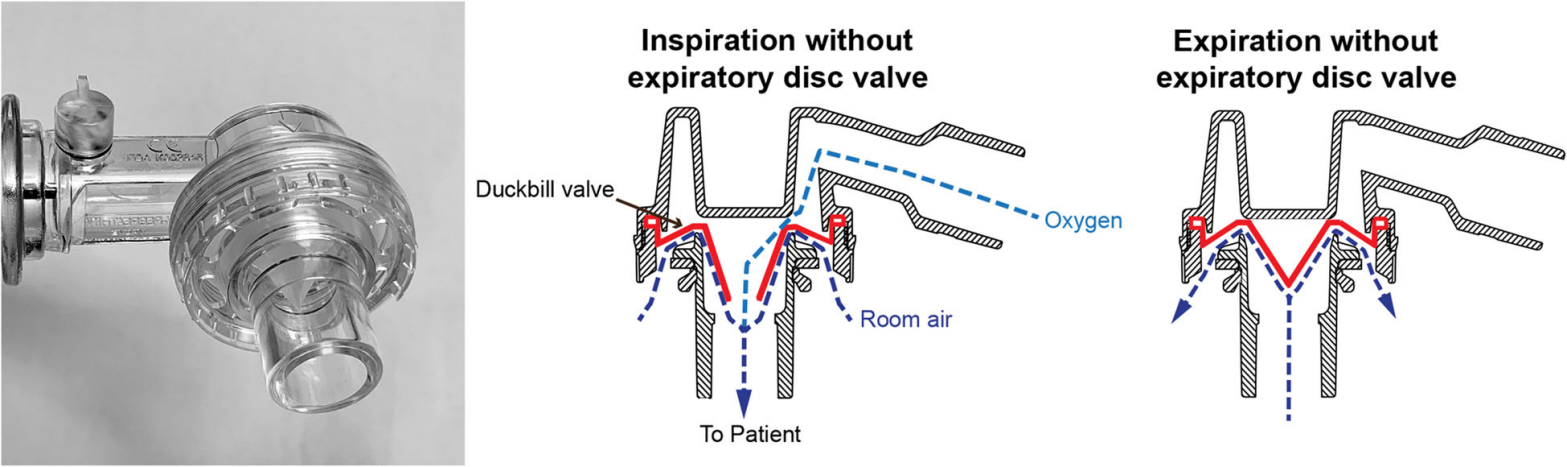
The duckbill valve without an expiratory valve (Intersurgical and O-Two). Drawing is used and modified with permission from www.laerdal.com

**Fig. 4:**
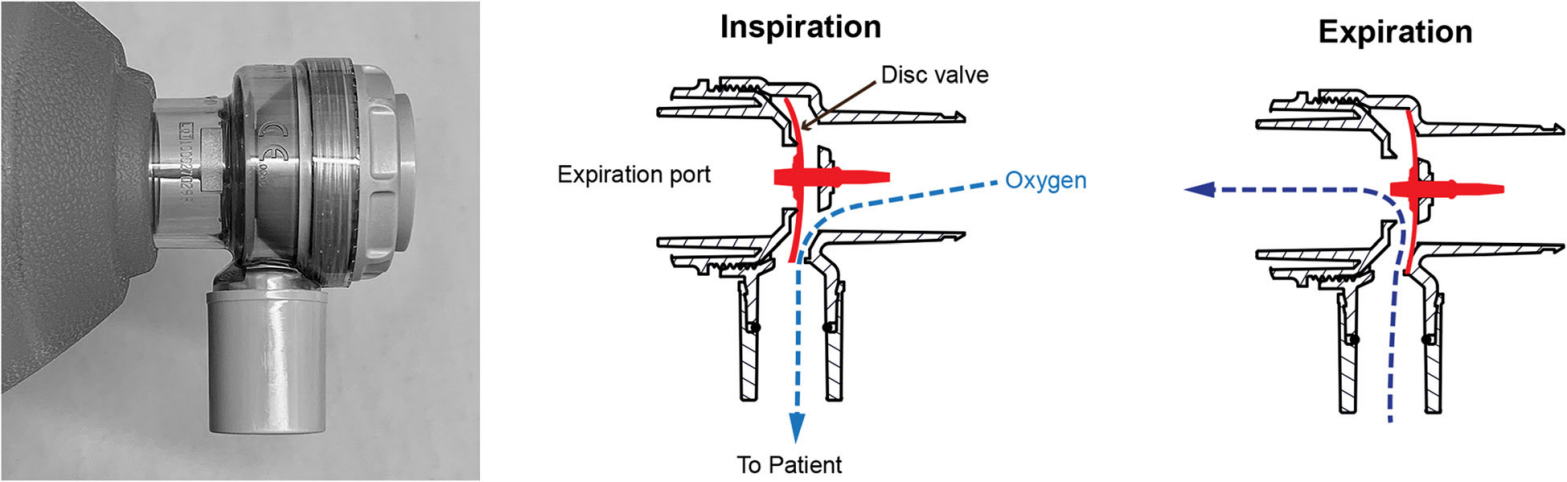
The disc valve (Ambu). Drawing is used and modified with permission from www.ambu.com

The two patient valve designs used in the tested models and most other available SIRBs are the duckbill valve and the disc valve (see Figs. 2, 3 and 4). The main reason for variations in FDO_2_ performance seems to be the presence of an expiratory valve. In our study, as well as in previous studies, the poor FDO_2_ performance was largely seen in devices with a *duckbill* design lacking a dedicated disc valve blocking the exhaust port during inspiration (see Fig. 3). This allows for air entrainment through the exhaust port during inspiration, resulting in a mixture of room air and oxygen being delivered to the patient.

In devices without an expiratory valve, the degree of air entrainment through the exhaust port will mainly depend on the opening pressure and flow resistance of the duckbill valve and the flow resistance of the exhaust port. Though this was not measured in our setup, a higher opening pressure and/or flow resistance of the duckbill valve will likely allow for more air entrainment through the low resistance opening of the exhaust port. If the expiratory valve does not seal properly it can allow for some entrainment of air. This has been shown previously [11] and might explain the smaller variation of FDO_2_ among the SIRBs *with* an expiratory valve that we tested.

**Fig. 5:**
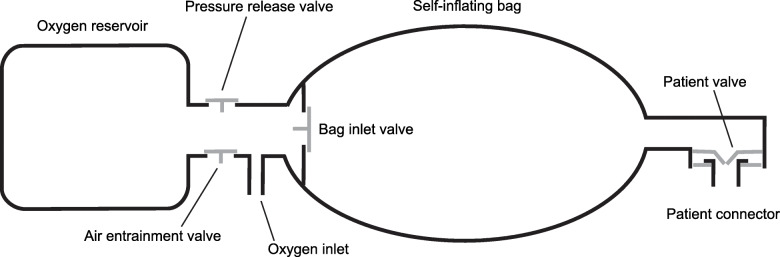
Cross-section drawing of a generic SIRB with a duckbill valve

The other valves in the SIRB add to the complexity (Fig. 5). The flow resistances and opening pressures of these valves determine forward flow pressures and the influence the oxygen fresh gas flow will have on the opening pressure of the patient valve. Increased oxygen fresh gas flow to the SIRB fills the reservoir and generates forward flow, which can lower the opening pressure of the inspiratory valve, resulting in less air entrainment at the patient end. Hence the oxygen fresh gas flow rate becomes more important in devices without a valve on the exhaust port. These factors are probably the main mechanisms behind the performance variation seen among duckbill SIRBs *without* an expiratory valve.

Adding a disc valve or a PEEP-valve to the exhaust port will markedly attenuate the entrainment of room air (not reported) and improve performance. This strongly supports that the lack of an expiratory valve is the main cause of the poor performance of some SIRBs. We have not found any explanation as to why some manufacturers have chosen to omit the expiratory valve in their SIRBs.

The *disc valve* (Fig. 4) on the other hand, has a different design where the inspiratory valve leaflet blocks the exhaust port during inspiration and thus prevents a significant entrainment of room air. To our knowledge there is no study that has shown any significant air entrainment in SIRBs with the disc valve design.

The poorest performer, the O-Two, has an extra flow limiting valve in the neck between the bag and the duckbill valve, in addition to lacking an expiratory valve. The ‘SMART BAG’ feature is proposed to limit high pressures and slow down positive pressure breaths if the bag is squeezed too hard or fast when supplying positive pressure ventilation. Even with this function disabled, as in our tests, it is likely that this valve adds resistance to forward flow, further raising the opening pressure of the duckbill valve during spontaneous breathing.

Previous data also indicates that breathing variables such as TV, RR, breath profile and the peak inspiratory flow rate (PIFR) can affect oxygen delivering capacity [11]. These patient factors interact with the reservoir, casing, valve constructions and oxygen fresh gas flow in a way that is not always predictable.

The oxygen fresh gas flow rate must always exceed the minute volume (MV) to have a theoretical chance of delivering an FDO_2_ of 1.0. In our test profile, we used a MV of 10 L/min and an oxygen fresh gas flow of 15 L/min to reduce the risk of a mismatch affecting the test results. PIFR can exceed MV by multiple times, making a reservoir mandatory on a SIRB when using an oxygen fresh gas flow rate lower than PIFR. The reservoir size ought to be larger than the TV to sustain a high FDO_2_. Flush rate oxygen fresh gas flow (30–60 L/min) matching the PIFR can reduce the need for a reservoir but this option is not always available and certainly not in the prehospital environment.

The pros and cons of disposable versus reusable SIRBs were not the focus of our study. It is worth noting though, that we have not found any SIRB performance studies reporting this problem with reusable devices. That said, there are disposable devices that perform just as well or even better than some reusable devices, e.g. Ambu in our study.

### Clinical implications

A predictable and constant FDO_2_ is crucial in supporting or preoxygenating spontaneously breathing critically ill patients. Using a SIRB that delivers a suboptimal oxygen flow in this setting can result in critical hypoxemia. We believe that a SIRB, with a verified oxygen flow, generally is the fall-back option for most operators to resolve many critical situations. This combination of circumstances can result in severe consequences.

The widespread knowledge gap about poor performing SIRBs can also lead to incorrect or conflicting conclusions from research. There are multiple examples where a single model of SIRB was used as a reference device in a comparative study between oxygen delivery devices [7–9, 15]. In a study from Robinson et al. [8] the EtO_2_ during preoxygenation of healthy volunteers were compared between a non-rebreather mask (NRM) and a SIRB (Intersurgical Adult Resuscitator) at an oxygen fresh gas flow of 10 L/min. The results showed that, while neither performed well, they produced comparable levels of EtO_2_. They concluded that the NRM might be a preferable approach of preoxygenation in that setting. Their choice of the Intersurgical Adult Resuscitator as benchmark was unfortunate and likely affected their results and conclusion. It might also have led to less than ideal practices in some services. Their study highlighted that many clinicians might take for granted that a SIRB with a tight seal mask achieves a near “gold standard” level of preoxygenation.

### Strengths and limitations

Mechanical simulations are different from human or animal experiments. This is a limitation, but we believe that our method reliably explores the capacity for oxygen delivery in a SIRB.

Our setup generated highly reproducible recordings with low variability and statistical significance at differences that were below what is clinically relevant. We tested three devices of each model and repeated the recording three times to minimize the impact of a malfunctioning SIRB. There is a limitation in that we used a single breathing profile and one level of oxygen flow. The extensive study by Mills et al. [11] included 27 combinations of breathing profiles and flow rates but our findings are in line with theirs. Additionally, they found large variations of FDO_2_ related to the different breathing profiles and oxygen flow rates. It is likely that using more challenging breath profiles and more oxygen fresh gas flow levels would have affected the SIRB performance in our study as well.

### Recommendations and future directions

We agree with previous researchers and urge manufacturers of underperforming SIRBs to either improve the spontaneous breathing performance or retract them from the market. Since there are both reusable and disposable SIRBs that perform well it is our opinion that a suboptimal SIRB has no place in healthcare today. Furthermore, we encourage all SIRB manufacturers to state their devices’ oxygen delivery performance in clinically relevant breathing scenarios during spontaneous breathing, in addition to positive pressure ventilation.

Further research areas in this field would be to make clinical studies to confirm or refute our findings. Another area would be to develop a SIRB with less imposed work of breathing while still maintaining a high oxygen delivery.

## Conclusions

Our study shows that there are Self-Inflating Resuscitation Bags on the market that underperform in oxygen delivery when used for spontaneous breathing. These devices might pose a serious threat to patients’ health if used in resuscitation and anaesthesia. It is our opinion that Self-Inflating Resuscitation Bags that do not provide sufficient oxygen delivery during spontaneous breathing should not be used until further clinical studies prove them safe.

## Data Availability

The datasets used and analysed during the current study are available from the corresponding author on reasonable request.

## Abbreviations

BVM: Bag Valve Mask
EtO_2_: End-tidal Oxygen Concentration
FDO_2_: Fraction of Delivered Oxygen
FiO_2_: Fraction of inspired Oxygen
MV: Minute Ventilation
NRM: Non-rebreather mask
PEEP: Positive End-Expiratory Pressure
PIFR: Peak Inspiratory Flow Rate
RR: Respiratory Rate
SIRB: Self-Inflating Resuscitation Bag
TV: Tidal Volume
